# Getting off the carousel: De-centring the curriculum in medical education

**DOI:** 10.1007/s40037-017-0373-x

**Published:** 2017-09-26

**Authors:** Cynthia R. Whitehead

**Affiliations:** The Wilson Centre, University of Toronto, Toronto, Ontario Canada

It is refreshing to see a research study examining the specificities and particularities of a change process in medical education. ‘Getting off the carousel: Exploring the wicked problem of curriculum reform’ by Hawick et al. does exactly that, providing the medical education community with a very interesting case study [1]. As Hawick et al. note, there is a commonly held belief that curriculum reform is linear, with more focus on outcomes than on sociocultural factors. This may well contribute to the fact that many reforms lead to little real change. Hawick et al. set out to study this issue by examining what happened in a UK medical school which changed its curriculum from a ‘traditional’ model (with the first two years being classroom-based and the final three years clinical) to a blended model that integrated clinical and basic science teaching and learning. The authors highlight the importance of context and values in shaping the change process they studied. They describe a multiplicity of views and voices from different stakeholders representing differing values that led to different definitions of ‘the problem’ being solved. The research team point to the roles of government and regulatory bodies in shaping how reforms were enacted, and identify the high degree of ambiguity inherent in a curriculum change process. In addition, they explore how power differentials and the values of individual leaders affected the reforms, revealing unintended consequences of the initiative.

The authors are to be commended for undertaking this study. Alas, studies like this are few and far between, which is a significant gap: our understanding of the real-life effects of curricular reforms could be significantly advanced if medical education researchers, in varying contexts, were to conduct similar work. As a colleague and I have argued elsewhere [2], the more comprehensive the reform initiative, the more those driving the reform should be required to design and carry out a rigorous program evaluation. There is no doubt that our field would be enriched, our science advanced, and our educational offerings more robust should we regularly evaluate the implementation of new initiatives.

Hawick et al. partially frame their work using the metaphor of a curricular carousel that I proposed some years ago in a paper entitled ‘Captive on a carousel: Discourses of ‘new’ in medical education 1910–2010’ [3]. In that article I suggested several factors that might contribute to the recurrence of reforms with limited change, including the possibility that significant changes to existing power structures may not be desired by those in control. To focus on changing the curriculum suggests that a solution to whatever is the ‘problem’ can be solved by tweaking the training of the next generation of learners, rather than contending with institutional structures and systems. Hawick et al., in their empirical study, however, note the effects on reform decisions by various institutional players, including the National Health Service. Moving from idealised views of the potential benefits of a new curriculum to the actual processes of implementation and their effects is an important shift in thinking. This shift involves a change from putting the curriculum at the centre of attention (in a way that makes it the primary object of focus) to contextualizing the curriculum. In so doing, we de-centre the curriculum.

The idea of curricular de-centring is not new. Kulasegaram et al. argue in ‘Cognition before curriculum: Rethinking the integration of basic science and clinical sciences’ [4] that our understandings of the cognitive processes of learners should shape the development of curricular content at a session level, instead of hoping that mapping out program or course level integration will result in an integrated curriculum. Building on the findings of Hawick et al., perhaps there are additional factors to foreground along with cognitive processes? A comprehensive analysis of all potential factors required to get off the curricular carousel is beyond the scope of this commentary, so I will touch on three I consider key: context, values and growth.

Hawick et al. highlight the importance of the context in which their curriculum reform took place. The medical education community has recognised the importance of context in medical education, largely through Hafferty’s extremely useful notion of the hidden curriculum [5]. This recognition, however, has not to date led to many specific studies of the contexts of curriculum (or other) reforms in medical education. Instead, there is a tendency to highlight the desired goal, be it a problem-based learning curriculum [6–9] simulation in the curriculum [10–12] or a competency-based curriculum [13–15]. More explicit and deliberate study of specific contexts of medical education will help our community better attend to the sociocultural, political, historical, and economic factors in which these various curricular reforms are implemented.

Hawick et al. also discuss the importance of the different values held by different stakeholders. Our field has not been terribly explicit about values to date. While educators regularly proffer platitudes about being socially responsible, ensuring that we are technologically up-to-date, or providing an efficient educational program [16–20], reformers are not often specific about the values that underpin rationales for the specific curricular changes proposed. Perhaps we should also expect educators to foreground values when undertaking curricular change?

In addition to putting context and values before curriculum, we might enhance our chances of getting off the curricular carousel by attending to Ursula Franklin’s incisive analysis in The Real World of Technology [21]. Franklin argues for a growth rather that a production model of education, articulating fundamental differences between the two. Growth occurs (rather than is made); growth can be supported in an appropriate environment; the job of educators is to find optimal conditions in which growth can occur, remembering that in any particular environment, an organism grows at its own rate [P. 20–21]. In a production model of education, in contrast, things are made in controlled and predictable ways, and context is unimportant. Franklin is unequivocal about which model fits education:

*Although we all know that a person’s growth in knowledge and discernment proceeds at an individual rate, schools and universities operate according to a production model. […] All of us who teach know that the magic moment when teaching turns into learning depends on the human setting and the quality and example of the teacher – on factors that relate to a general environment of growth rather than on any design parameters set down externally. If there ever was a growth process, if there ever was a holistic process, a process that cannot be divided into rigid predetermined steps, it is education [P. 22–23].*

When Franklin wrote this in 1990, she noted that taking on the production model of education would be difficult, as ‘production-based models and metaphors are already so deeply rooted in our social and emotional fabric that it becomes almost sacrilege to question them.’ [P. 26] Nevertheless, question we must. More than 25 years after Franklin’s warning, production models have become even more entrenched in medical education than they were when she initially cautioned against them. Mechanistic rather than organic models dominate our current education landscape. Production models of education foreground efficiency, and seek cost-effective inputs and outputs [P. 169]. In a production model, so long as students pass their exams and meet competency checklists, the nurturing of attributes such as tolerance and patience is less relevant. A growth model, in contrast, foregrounds the learning environments in which the ‘products’ of education are embedded. By attending to the learning environment, that space is shaped to encourage aspects of social learning such as cohesion, trust, anger management and cooperation. [P. 172]. As Franklin observes, in a production model, while ‘the pool of information available to the students may increase, the pool of available understanding may not.’ [P. 171].

To get off the curricular carousel, medical educators should consider whether our reliance on production models is shaping our curricular reforms in desirable ways. Should the medical education community see places in which production models fall short, perhaps our field could – more than a generation after Franklin’s analysis – shift towards growth models of education. What might medical education become if we not only put cognition before curriculum, but also considered context, values, and growth before curriculum? What might such a curricular de-centring lead to? Very likely, the change would be radical.

For over a century, medical educators have been engaged in a curriculum war, with basic scientists battling with bioethicists, nephrologists sparring with neurosurgeons, and technical skills teaching vying with communication skills for curricular time. Might it be time to draw this Hundred Years War to a close? Medical educators could do well to consider the possibilities that might come from curricular peace. By de-centring the curriculum and foregrounding growth models of education, while focussing on cognition, context, and values, medical educators might perhaps start to re-imagine the training of medical healers in our clinically complex, technologically changing, and societally challenging times.

Are we as a community of educators ready to try to get off the curricular carousel? If we de-centre the curriculum we should be able, in the specific contexts in which we work (together with teachers, learners, and patients), to find ways towards less hierarchical, more inclusive models and structures of medical education. While the medical curriculum is mighty, medical educators can – if we choose – stop paying the curriculum quite so much homage and let it take its rightful place as a mere tool which we can and should use to best advantage.
